# The instant effect of embodiment *via* mirror visual feedback on electroencephalogram-based brain connectivity changes: A pilot study

**DOI:** 10.3389/fnins.2023.1138406

**Published:** 2023-03-20

**Authors:** Li Ding, Qiang Sun, Ning Jiang, Jiayuan He, Jie Jia

**Affiliations:** ^1^Department of Rehabilitation Medicine, Huashan Hospital, Fudan University, Shanghai, China; ^2^The National Clinical Research Center for Aging and Medicine, Huashan Hospital, Fudan University, Shanghai, China; ^3^National Clinical Research Center for Geriatrics, West China Hospital, Sichuan University, Sichuan, China; ^4^Med-X Center for Manufacturing, Sichuan University, Sichuan, China

**Keywords:** mirror visual feedback, embodiment perception, network, electroencephalogram, rehabilitation, vibrotactile simulation

## Abstract

The therapeutic efficacy of mirror visual feedback (MVF) is attributed to the perception of embodiment. This study intends to investigate the instantaneous effect of embodiment on brain connectivity. Twelve healthy subjects were required to clench and open their non-dominant hands and keep the dominant hands still during two experimental sessions. In the first session, the dominant hand was covered and no MVF was applied, named the sham-MVF condition. Random vibrotactile stimulations were applied to the non-dominant hand with MVF in the subsequent session. Subjects were asked to pedal while having embodiment perception during motor tasks. As suggested by previous findings, trials of no vibration and continuous vibration were selected for this study, named the condition of MVF and vt-MVF. EEG signals were recorded and the alterations in brain connectivity were analyzed. The average node degrees of sham-MVF, MVF, and vt-MVF conditions were largely different in the alpha band (9.94, 11.19, and 17.37, respectively). Further analyses showed the MVF and vt-MVF had more nodes with a significantly large degree, which mainly occurred in the central and the visual stream involved regions. Results of network metrics showed a significant increment of local and global efficiency, and a reduction of characteristic path length for the vt-MVF condition in the alpha and beta bands compared to sham-MVF, and in the alpha band compared to MVF. Similar trends were found for MVF condition in the beta band compared to sham-MVF. Moreover, significant leftward asymmetry of global efficiency and rightward asymmetry of characteristic path length was reported in the vt-MVF condition in the beta band. These results indicated a positive impact of embodiment on network connectivity and neural communication efficiency, which reflected the potential mechanisms of MVF for new insight into neural modulation.

## 1. Introduction

Mirror visual feedback (MVF) is a non-invasive priming technique that has been widely adopted in neurorehabilitation. Reviews have demonstrated the therapeutic effects of MVF on improving motor recovery and daily living ability after stroke, especially for upper limb function (Rothgangel et al., 2011; Pollock et al., 2014; Thieme et al., 2018). A plain mirror placed the moving non between the upper limbs reflects -affected hand and the reflection produces the embodiment perception of the affected hand, which has effects on the recovery of motor functions after impairments. To present an optimal use in the clinic, lots of studies have investigated the correlated potential neuro-mechanisms of MVF, including activations of widely distributed brain regions, normalization of hemisphere asymmetry, and brain activity modulation (Michielsen et al., 2011a,b; Saleh et al., 2014; Rossiter et al., 2015; Chang et al., 2017). Observed activation of motor cortical regions *via* EEG and fMRI, such as the primary motor cortex and supplementary motor area (SMA), is one of the direct and instant effects of MVF (Michielsen et al., 2011a; Debnath and Franz, 2016; Ding et al., 2020a). Moreover, increased activities of the postcentral gyrus, frontal-parietal areas, posterior parietal cortex, precuneus, visual cortex, and dorsolateral prefrontal cortex are also observed during MVF (Fink et al., 1999; Matthys et al., 2009; Michielsen et al., 2011b; Arya, 2016; Rizzo et al., 2022), which are responsible for sensorimotor coordination, visuomotor transformation, and visual attention. Regarding the long-term effects of MVF, studies showed facilitating corticospinal outputs and restoring hemispheric balance after MVF intervention (Michielsen et al., 2011a; Lappchen et al., 2012). Brain connectivity is regarded as a primary contributor to brain reorganization, which has been described using graph theory (Caliandro et al., 2017). However, only a few studies investigated the underlying mechanisms of MVF from the perspective of brain connectivity (Hamzei et al., 2012; Rjosk et al., 2017; Saleh et al., 2017; Ding et al., 2019). Hamzei et al. (2012) reported an increased interaction between the premotor region and SMA after MVF training in healthy subjects. Rjosk et al. (2017) investigated the improved performance induced by MVF and concluded that it was the result of enhanced connections between perceptual and motor cortical regions, including the primary sensorimotor cortex, visual cortex, and anterior intraparietal sulcus. Furthermore, a dynamic causal modelling study showed an effective connection between the primary cortex and the contralateral parietal cortex (Saleh et al., 2017), which suggested the MVF-based motor activation was driven by the contralateral parietal cortex. In our previous study, increased network segregations in the visual, somatosensory, and motor areas were observed, which suggested enhanced communication efficiency after MVF intervention in stroke patients (Ding et al., 2019). To the best of our knowledge, rare studies explored the instant effects of MVF on network topology and communication efficiency using EEG for new insight, which outperformed MRI on temporal resolution. Thus, we intended to explore the instantaneous effect of embodiment *via* MVF on network connectivity and the efficiency of information transmission.

Embodiment is a subjective sense of self, comprising body ownership, location, agency, and deafference (Blanke et al., 2015). Studies presented that the therapeutic efficacy of MVF might be attributed to the perception of embodiment, which could affect the sensorimotor activity and multisensory integration (Michielsen et al., 2011b; Medina et al., 2015; Ding et al., 2019). Our previous studies found multisensory inputs could strengthen embodiment perception during MVF and observed correlative enhanced motor cortical activation in healthy subjects (Ding et al., 2020a,b). Moreover, a slightly higher weighted clustering coefficient and shorter weighted path length were obtained in our prior study when receiving MVF with continuous vibrotactile simulation (Ding et al., 2020a). Therefore, we hypothesized a potential influence of embodiment intensity on network topology, which might contribute to further exploring its effect on brain connectivity changes.

In this study, no MVF, pure MVF, and MVF with vibrotactile stimulation were employed to induce different degrees of embodiment perception and the related instant effects on the EEG-based brain network were investigated. As more in-depth analyses of our previous experiment, we aimed to provide more tentative evidence on the potential mechanisms of MVF for new insight from the brain connectivity perspective and further exploration of neural modulation. Moreover, part of the dataset has been used in our previous study and re-analyzed in this present study (Ding et al., 2020a).

## 2. Materials and methods

### 2.1. Subjects

Twelve healthy subjects with an average age of 25 years (four females and eight males) were recruited for the experiment. None of these subjects experienced any MVF experiment before. Prior to the experiment, all subjects were informed, agreed, and signed the consent form approved by the Research Ethics Committee of the University of Waterloo (ORE# 22900).

### 2.2. Experimental paradigm

**FIGURE 1:**
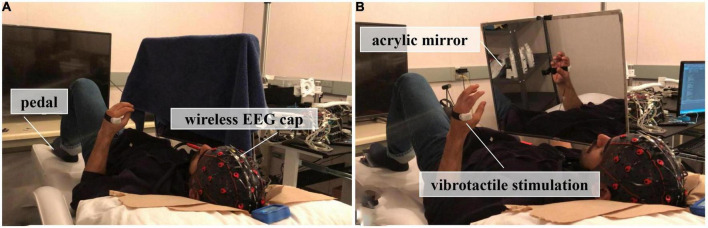
Experimental setup, **(A)** The first session, where no mirror visual feedback (MVF) was applied; **(B)** The second session, where subjects received MVF or MVF combined with vibrotactile stimulation.

In the experiment, an acrylic mirror (40 cm by 50 cm) was suspended on a holder and placed over the chest of a lying subject in a sagittal plane, see Figure 1. Subjects were required to lie down on a bed, clench their fists, and open their hands using their non-dominant hands (two out of the twelve subjects were left-handed) at the pace of 1 Hz on the reflecting side of the mirror, place the dominant hands in a similar position as the non-dominant side and keep static behind the mirror. Moreover, a pedal was set under the non-dominant foot, and subjects were asked to press on the pedal for a very short time as soon as they experienced the sense of embodiment from the dominant side *via* mirror reflection, such as the feeling that he/she could move the reflection without moving the dominant hand or the reflection was part of his/her body.

**FIGURE 2:**
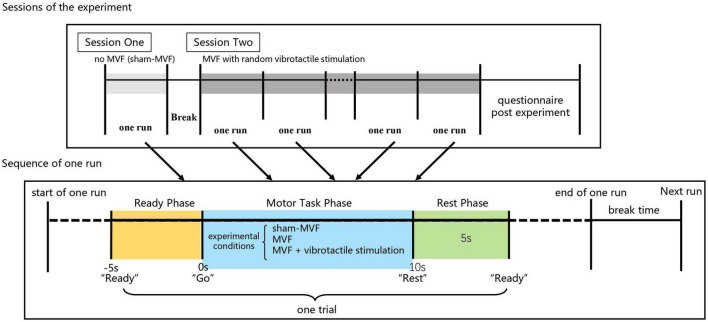
Experimental paradigm and sequence of events in each trial.

This experiment comprised two sessions, which was illustrated in Figure 2. In the first session, subjects performed 30 trials of hand motor tasks (non-dominant hand clenching and opening while dominant hand keeping still) without MVF and vibrotactile stimulation, which was defined as the condition of sham-MVF, see Figure 1A. In the subsequent session, subjects conducted the same hand motor task with MVF while vibrotactile stimulations were applied randomly, see Figure 1B. The vibrotactile stimulation was provided *via* a liner resonance actuator (type C10–100, Precision Microdrivers Ltd., London, England) at the first interosseous dorsal muscle tendon of the non-dominant hand. In this session, subjects performed 30 trials (as one run) for a total of 180 trials (six runs) and rested between trials. Each run contained 10 trials for each type of vibration that appeared randomly. The sequence of events in each trial for these two sessions was the same as illustrated in our previous study, including a ready phase, motor task phase, and rest phase (see Figure 2). In the previous study, enhanced embodiment was obtained while subjects received MVF combining vibrotactile stimulation (continuous and intermittent), according to the shorter pedal time latency and a higher score on the embodiment questionnaire (Ding et al., 2020a). Moreover, a stronger power desynchronization in the dominant hand area was found in the alpha and beta bands with an enhanced embodiment. However, around the parietal-occipital region, a relatively stronger power desynchronization was observed under MVF and MVF combined with continuous vibrotactile stimulation than the intermittent one in the both frequency bands (Ding et al., 2020a). Thus, trials of no vibration and continuous vibration were selected for the analyses in this study, which were defined as the condition of MVF and vt-MVF, respectively. Moreover, these data have been used in our previous study and re-analyzed in this study for further exploration (Ding et al., 2020a). EEG signals were recorded continuously in the experiment.

### 2.3. EEG signal acquisition and preprocessing

A 32-channel wireless EEG recording equipment (g.Nautilus, g.tec, Austria) was used in the experiment. Electrodes were placed at Fp1, Fp2, AF3, AF4, F7, F3, Fz, F4, F8, FC5, FC1, FC2, FC4, T7, C3, Cz, C4, T8, CP5, CP1, CP2, CP6, P7, P3, Pz, P4, P8, PO7, PO3, PO4, PO8, and Oz with their arrangements followed the extended 10–20 system. The reference and ground electrodes were placed on the right ear lobe and the forehead, respectively. EEG signals were recorded at a 250 Hz sampling rate and notch filtered the power line noise at 60 Hz. Raw data were cleaned offline using MATLAB (MathWorks, Inc., version 2021a) toolbox EEGLAB (Delorme and Makeig, 2004). First, EEG signals were visually inspected and trials with unexpected experimental artifacts, including large drifts and spikes, were removed. After that, independent component analysis (learning rate: 0.001; max number of interactions: 512; the other settings are in default from EEGLAB) was employed in the remaining trials to remove eye movement and ECG, etc., artifacts.

This study analyzed EEG signals of three conditions, including sham-MVF, MVF, and vt-MVF, in four frequency bands, including theta (4–8 Hz), alpha (8–13 Hz), beta (13–30 Hz), and gamma (low gamma band 30–40 Hz). The 4 s data after the subject pedaled (perceiving embodiment) was analyzed for the condition of MVF and vt-MVF. For the sham-MVF condition, as the subject doesn’t need to respond to their embodiment feeling, to better reflect their brain activity on account of response latency, we empirically choose the middle 4 s (4–8 s after the “Go” cue) data for analysis. Then, all segmented data were filtered into the frequency band of interest using a Hamming windowed sinc FIR filter (*pop_eegfiltnew()* function in EEGLAB). Note that, after removing noise-contaminated trials, there were an average of 22 trials per subject being remained for analysis of the sham-MVF condition. For the MVF and vt-MVF conditions, only trials with embodiment perception remained, which resulted in an average of 33.1 and 39.2 trials per subject, respectively.

### 2.4. Construction of functional connectivity networks

A brain network consisted of multiple nodes (EEG electrodes) and edges (connectivity strength between two nodes). In this study, a 32-node brain network was constructed with each node representing one specific electrode. To estimate the functional connectivity of pairwise nodes, the phase lag index (PLI), which measures the asymmetry of the distribution of phase differences between two signals while eliminating volume conduction effects (Stam et al., 2007), was applied. Given two signals *x*(*t*) and *y*(*t*) from pairwise nodes, together with their Hilbert transform ${}_{\tilde{x}\left(t\right)}$ and ${}_{\tilde{y}\left(t\right)}$, the analytical signal _*z_x_(t)*_ and _*z_y_(t)*_ can be formulated as:


(1) $$\begin{array}{c} z_{x}\,\left(t\right)=x\,\left(t\right)+i\,\tilde{x}\,\left(t\right) \end{array}$$

(2) $$\begin{array}{c} z_{y}\,\left(t\right)=y\,\left(t\right)+i\,\tilde{y}\,\left(t\right) \end{array}$$

with


(3) $$\begin{array}{c} \tilde{x}\,\left(t\right)=\frac{1}{\pi }\,P\,V\,\int_{-\infty }^{+\infty }\frac{x\,\left(\tau \right)}{t-\tau }\,d\,\left(\tau \right) \end{array}$$

(4) $$\begin{array}{c} \tilde{y}\,\left(t\right)=\frac{1}{\pi }\,P\,V\,\int_{-\infty }^{+\infty }\frac{y\,\left(\tau \right)}{t-\tau }\,d\,\left(\tau \right) \end{array}$$

where *PV* refers to the Cauchy principal value.

Then, the instantaneous phases _Φ_x_(*t*)_ and _Φ_y_(*t*)_ of *x*(*t*) and *y*(*t*) can be calculated as:


(5) $$\begin{array}{c} {\text{Φ}}_{x}\,\left(t\right)=a\,r\,c\,t\,a\,n\,\frac{\tilde{x}\,\left(t\right)}{x\,\left(t\right)} \end{array}$$

(6) $$\begin{array}{c} {\text{Φ}}_{y}\,\left(t\right)=a\,r\,c\,t\,a\,n\,\frac{\tilde{y}\,\left(t\right)}{y\,\left(t\right)} \end{array}$$

Finally, we can obtain the PLI value of the pairwise nodes:


(7) $$\begin{array}{c} P\,L\,I=\left|\frac{1}{N_{s}}\,\sum_{k=1}^{N_{s}}\text{sign}\,\left[\sin\Delta \,\text{Φ}\,\left(t_{k}\right)\right]\right| \end{array}$$

where *N_s_* denotes the sample number, _Φ(*t_k_*)_ denotes the phase differences between _Φ_*x*_(*t*)_ and _Φ_*y*_(*t*)_ at _*t_*k*_*_. Note that the PLI value ranges between 0 and 1 with 0 indicating either no or instantaneous coupling and 1 indicating perfect phase locking (Stam et al., 2007).

After calculating the PLI value of all possible pairwise nodes, a 32 × 32 weighted adjacency matrix was yielded for each trial. In this study, we aimed to analyze brain network differences of different experimental conditions on each frequency band in an individual and grouped fashion. Specifically, the individual functional connectivity matrix of each subject on each frequency band and experimental condition was calculated by averaging the adjacency matrices across all trials, while the grouped functional connectivity matrix was calculated by averaging the individual functional connectivity matrices across subjects.

### 2.5. Network binarization

A critical step in network construction is to exclude the weak and spurious connections within the graph. In this study, we employed the Cluster-Span Threshold (CST) method for unbiased network binarization (Smith et al., 2015, 2016). CST determines the optimal proportional threshold on the condition that the network topology achieves balance in segregation and integration. Given that triples, formed by two neighboring nodes sharing connections with one common node, play a key role in graph analysis, network integration, and segregation can be represented by clustering (or closed) and spanning (or open) triples, respectively. Therefore, the alternative definition of CST selects the proportional threshold for which the ratio of clustering triples to spanning triples is balanced. This situation happens when the global clustering coefficient (*CC*_glob_, see its definition in the next section) of the binarised network equals 0.5.

In this study, we searched the threshold value across the sparsity level from 10 to 100% with an interval of 1%. Sparsity level is defined as the proportion of remaining edges to all possible edges of a network. Due to the discrete nature of a binarized network, we technically took the CST as the proportional threshold at which the *CC*_glob_ was closest to 0.5.

### 2.6. Network metrics

Multiple measures can be used to characterize network connectivity, as suggested by the review (Rubinov and Sporns, 2010). Based on grouped functional connectivity matrices, we first computed the degree of each node, which represents the importance of a node within the network, as it is a commonly used measure of centrality. In such a case, we aimed to investigate the distribution difference of functional connectivity between experimental conditions on different brain regions. Furthermore, we investigated the network segregation and integration differences between experimental conditions *via* individual functional connectivity matrices. Here, the clustering coefficient (*CC*) and local efficiency (*LE*) were adopted as segregation measures, and the characteristic path length (*CPL*) and global efficiency (*GE*) were adopted as integration measures. All these network metrics were calculated based on the brain connectivity toolbox (BCT),^1^ and their mathematical definition sees follows.

Assume that a network has *n* nodes, and all nodes form a set *N*. Moreover, the connection status between node *i* and *j* is denoted as _*a_ij_*_ (_*a_ij_ =1*_ if a link exists between *i* and *j*, otherwise, _*a_ij_ =0*_), the shortest path length between node *i* and *j* is denoted as _*d_ij_*_, and the length of the shortest path between node *j* and *h* that contains only the neighbors of node *i* is denoted as *_*d_jh_(N_i_)*_*. Then, the degree of a given node *i* can be obtained as the number of connected links:


(8) $$\begin{array}{c} k_{i}=\sum_{j\in N}a_{i\,j} \end{array}$$

The larger the node degree is, the more important this node is within the network. Besides, *CC*, *LE*, *CPL*, and *GE* of the network are formulated as:


(9) $$\begin{array}{c} C\,C=\frac{1}{n}\,\sum_{i\in N}C\,C_{\mathrm{node},i}=\frac{1}{n}\,\sum_{i\in N}\frac{\sum_{j,h\in N}a_{i\,j}\,a_{i\,h}\,a_{j\,h}}{k_{i}\,\left(k_{i}-1\right)} \end{array}$$

(10) $$\begin{array}{c} L\,E=\frac{1}{n}\,\sum_{i\in N}\frac{\sum_{j,h\in N,j\neq i}a_{i\,j}\,a_{i\,h}\,{\left[d_{j\,h}\,\left(N_{i}\right)\right]}^{-1}}{k_{i}\,\left(k_{i}-1\right)} \end{array}$$

(11) $$\begin{array}{c} C\,P\,L=\frac{n\,\left(n-1\right)}{\sum_{i\in N}\sum_{j\in N,j\neq i}d_{i\,j}^{-1}} \end{array}$$

(12) $$\begin{array}{c} G\,E=\frac{\sum_{i\in N}\sum_{j\in N,j\neq i}d_{i\,j}^{-1}}{n\,\left(n-1\right)} \end{array}$$

where*CC*_*node*,*i*_ represent the clustering coefficient of node *i*. The upper mentioned *CC*_glob_ is defined as the summation of *CC*_node_. Note that to avoid infinite value caused by disconnected pairs of nodes, in this study, *CPL* is defined as the harmonic mean of the shortest path length as recommended by Newman (2003), which is also the reciprocal of *GE*. Moreover, as hubs are vital components for network efficient communication and reliance (Hosseini et al., 2012), we consider a node as a hub if its degree is one standard deviation higher than the mean network degree (Bernhardt et al., 2011).

### 2.7. Hemispheric asymmetry analysis

Although many studies reported the ability of MVF on balancing hemisphere asymmetry and modulating hemispheric activation (Rossiter et al., 2015; Bartur et al., 2018; Rohafza et al., 2019; Zhang and Fong, 2019), its effect on shifting network metrics is rarely reported. Thus, as an additional measure, the hemispheric asymmetry in metrics was analyzed for future study. The hemispheric asymmetry of connectivity patterns was analyzed *via* the laterality index (LI) (Hassanin et al., 2021). In this study, the LI value is defined as follows:


(13) $$\begin{array}{c} L\,I=\frac{N\,M_{r}-N\,M_{l}}{N\,M_{r}+N\,M_{l}} \end{array}$$

where *NM*_*r*_ and *NM*_*l*_ indicate the network metrics of the right and left sub-network, respectively. To be specific, two groups of channels (i.e., right group: FP2, AF4, F8, F4, FC6, FC2, T8, C4, CP6, CP2, P8, P4, PO8, PO4; left group: FP1, AF3, F7, F3, FC5, FC1, T7, C3, CP5, CP1, P7, P3, PO7, PO3) were selected out of the overall recording channels. Then, the sub-network corresponding to each group was constructed and used for the regional clustering coefficient, characteristic path length, local efficiency, and global efficiency estimations. The LI value was finally computed based on these regional network metrics. In the study, the LI values ranged from −1 to 1, with negative values indicating the left hemisphere (representing the dominant hand) and vice versa.

### 2.8. Statistical analysis

We performed one-way repeated measures analysis of variance (ANOVA) to test the effects of experimental conditions on segregation and integration measures. The conditions of sham-MVF, MVF, and vt-MVF were the three levels of the single factor. Post-hoc multiple comparisons were conducted with Bonferroni correction. Besides, paired *t*-test was used to test the significant node degree difference between different experimental conditions, as well as the LI value difference between the left and right brain regions on each experimental condition. Note that the normality of parameter distribution was first determined by Shapiro-Wilk’s test, and the homogeneity of parameter variance was checked by Bartlett’s test. All tests had a significance level of 0.05. The script used for statistical analysis was developed based on built-in functions in MATLAB.

## 3. Results

### 3.1. Scalp network topology

**FIGURE 3:**
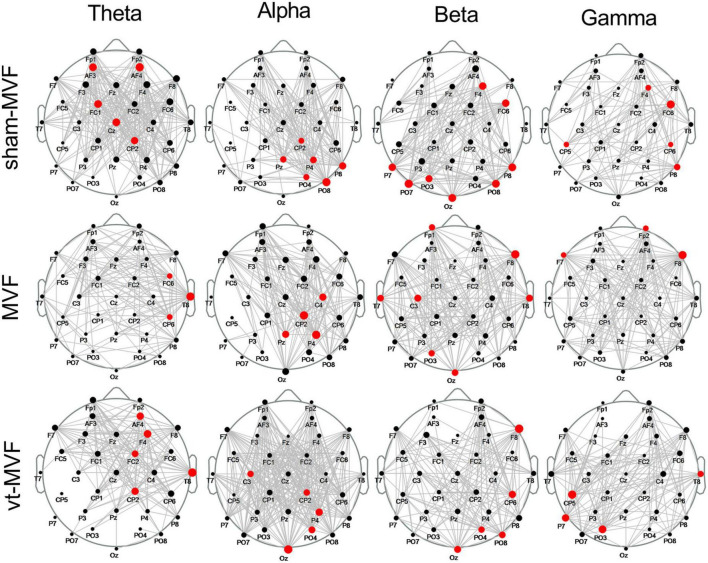
Scalp network topology of the sham-mirror visual feedback (MVF), MVF, and vt-MVF experimental condition in theta (4–8 Hz), alpha (8–13 Hz), beta (13–30 Hz), and gamma (30–40 Hz) frequency band. The bigger dot on each node (electrode location) indicates the larger node degree within this network. Note that the hubs are marked in red.

The scalp network topology of the sham-MVF, MVF, and vt-MVF experimental conditions after CST binarization was shown in Figure 3. Besides, the binarization threshold of node degree was also presented in Table 1. As can be seen, MVF and vt-MVF conditions enjoyed more prominent connectivity compared to the sham-MVF condition in the alpha and gamma bands. Specifically, the connectivity was largely different in the alpha band, where the average node degrees of the sham-MVF, MVF, and vt-MVF conditions were 9.94, 11.19, and 17.37, respectively. Moreover, only the MVF condition showed more dense connectivity compared to the sham-MVF in the beta band. In the theta band, the sham-MVF condition had more dense connectivity compared to MVF and vt-MVF conditions. To be specific, the average node degree of the sham-MVF condition was 14.87, which outperformed that of the conditions of MVF (9.31) and vt-MVF (9.62). Moreover, the network hubs were distributed differently under the three experimental conditions in each frequency band without distinct patterns in the meantime.

**TABLE 1:** Binarization threshold of brain networks in different frequency bands and the corresponding node degree.

	Theta	Alpha	Beta	Gamma
sham-MVF	14.87 (48%)	9.93 (32%)	10.87 (35%)	7.75 (25%)
MVF	9.31 (30%)	11.19 (36%)	12.37 (40%)	10.25 (33%)
vt-MVF	9.62 (31%)	17.37 (56%)	10.56 (34%)	9.62 (31%)

Data are formatted as node degree (binarization threshold).

**FIGURE 4:**
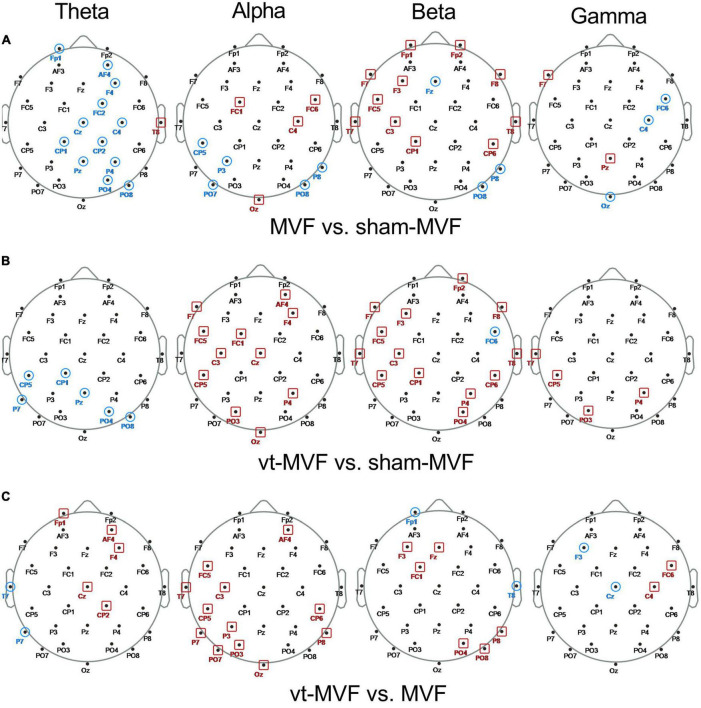
Scalp topological differences between **(A)** mirror visual feedback (MVF) and sham-MVF, **(B)** vt-MVF and sham-MVF, and **(C)** vt-MVF and MVF experimental conditions in different frequency bands (from left to right in each row corresponding to theta, alpha, beta, and gamma frequency band, respectively). Taking the condition pair MVF vs. sham-MVF as an example, the node marked with a red square denoted that the node degree of MVF on this location was significantly larger (*p* < 0.05) than that of sham-MVF, while the node marked with a blue circle denoted the opposite case.

To further explore the topological differences within the three experimental conditions, the node degree differences between different pairs of conditions were statistically investigated. As shown in Figure 4A, the sham-MVF condition had more nodes with a significantly larger degree than the MVF condition in the theta band on the middle brain regions (Frontal, Central, Central-Parietal, Parietal, and Parietal-Occipital regions), while in the alpha and the beta bands, the number of nodes with a significantly larger degree of the MVF condition increased (mainly distributed around Frontal, Central, Central-Parietal regions). Regarding the comparison between conditions of vt-MVF and sham-MVF (see Figure 4B), the vt-MVF condition had almost all nodes with a significantly larger degree in the alpha and the beta bands, which mainly located in the left Frontal-Central, left Central, Central-Parietal, Parietal, and Parietal-Occipital regions. However, in the theta band, the nodes with a significantly larger degree were shown in the condition of sham-MVF and mainly located in the left Central-Parietal, left Parietal, and right Parietal-Occipital regions. As demonstrated in Figure 4C, the vt-MVF condition had more nodes with a significantly larger degree compared to the MVF condition in the theta, alpha, and beta bands, especially in the alpha band and located in the left Frontal-Central, left Central, left Central-Parietal, left Parietal-Occipital regions. Furthermore, a few nodes with statistical differences were obtained *via* the comparison among three experimental conditions in the gamma band in this study.

### 3.2. Alteration of network metrics

One-way repeated measures ANOVA demonstrated significant interactions of experimental conditions on CPL in the theta [F(2, 22) = 4.4592, *p* = 0.0237], alpha [F(2, 22) = 5.8564, *p* = 0.0055], and beta [F(2, 22) = 5.9490, *p* = 0.0086] bands, LE in the alpha [F(2, 22) = 6.6530, *p* = 0.0021] and beta [F(2, 22) = 8.0101, *p* = 0.0024] bands, GE in the theta [F(2, 22) = 4.4727, *p* = 0.0234], alpha [F(2, 22) = 6.7632, *p* = 0.0051], and beta [F(2, 22) = 6.4428, *p* = 0.0063] bands, respectively.

**FIGURE 5:**
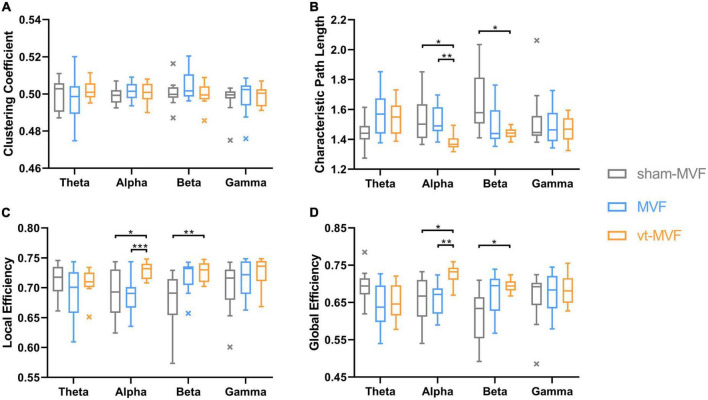
Network metrics, including **(A)** clustering coefficient, **(B)** characteristic path length, **(C)** local efficiency, and **(D)** global efficiency, of the three experimental conditions in different frequency bands. The central line in these boxplots indicates the median and the cross markers are the outliers. The asterisk indicates a significant difference between the two conditions (*0.01 < *p* < 0.05, **0.001 < *p* < 0.01, ***p < 0.001).

The results of post-hoc multiple comparisons were shown in Figure 5. According to Figures 5B, D, the vt-MVF condition showed significant increments of LE and GE, and significant reductions of CPL in the alpha band, compared to both the sham-MVF and MVF condition, which indicated an enhanced functional integration and segregation *via* the combination of vibrotactile stimulation. In the beta band, there were a significant reduction of CPL and an increment of LE and GE of the vt-MVF condition compared to that of the sham-MVF condition. Moreover, a similar trend could be found when comparing the MVF to the sham-MVF condition in the beta band, although without significant differences.

### 3.3. Hemispheric asymmetry

Table 2 showed the LI of the CC, CPL, LE, and GE under sham-MVF, MVF, and vt-MVF experimental conditions in different frequency bands. A significant leftward asymmetry of GE and rightward asymmetry of CPL were obtained in the condition of vt-MVF in the beta band, which indicated the improved general efficiency of brain neural communication in the left hemisphere (dominant hand). Besides, CC in the gamma band revealed a significant rightward asymmetry in the condition of vt-MVF, which suggested increased segregation of the right hemisphere. Moreover, a leftward asymmetry of LE in the gamma band was shown in the condition of sham-MVF.

**TABLE 2:** Laterality index (mean ± standard deviation) of the sham-MVF, MVF, and vt-MVF experimental conditions in different frequency bands.

Metric	Condition	Frequency band			
		Theta	Alpha	Beta	Gamma
*CC*	sham-MVF	−0.0003 ± 0.0073	−0.0071 ± 0.0134	0.0021 ± 0.0256	−0.0065 ± 0.0116
	MVF	0.0043 ± 0.0121	−0.0097 ± 0.0148	−0.0019 ± 0.0083	−0.0007 ± 0.0172
	vt-MVF	−0.0025 ± 0.0209	−0.0045 ± 0.0154	0.0038 ± 0.0099	**0.0066 ± 0.0095**
*CPL*	sham-MVF	−0.0336 ± 0.0886	−0.0025 ± 0.0638	−0.0289 ± 0.0962	−0.0044 ± 0.0357
	MVF	−0.0151 ± 0.1062	0.0068 ± 0.1306	0.0337 ± 0.0959	0.0158 ± 0.0657
	vt-MVF	−0.0198 ± 0.0477	−0.0133 ± 0.1023	**0.0399 ± 0.0392**	0.0069 ± 0.0789
*LE*	sham-MVF	0.0131 ± 0.0577	0 ± 0.0546	0.0055 ± 0.0213	**−0.0261 ± 0.0302**
	MVF	0.0199 ± 0.0532	−0.0077 ± 0.0506	−0.0254 ± 0.0622	−0.0125 ± 0.0503
	vt-MVF	−0.0057 ± 0.044	−0.0042 ± 0.0615	−0.0119 ± 0.0325	0.0063 ± 0.0483
*GE*	sham-MVF	0.0336 ± 0.0886	0.0025 ± 0.0638	0.0289 ± 0.0962	0.0044 ± 0.0357
	MVF	0.0151 ± 0.1062	−0.0068 ± 0.1306	−0.0337 ± 0.0959	−0.0158 ± 0.0657
	vt-MVF	0.0198 ± 0.0477	0.0133 ± 0.1023	**−0.0399 ± 0.0392**	−0.0069 ± 0.0789

Bold values indicated statistically significant (*p* < 0.05) laterality indexes, i.e., left and right sub-networks had a significant difference in network metrics.

## 4. Discussion

This study reveals a positive impact of the perception of embodiment on network connectivity and neural communication efficiency in healthy subjects. Moreover, the present study also provides tentative evidence that a stronger perception of embodiment induced *via* the combination of mirror visual and vibrotactile stimulus could enhance neural communication efficiency and generate a shift in global brain network efficiency toward the hemisphere of the dominant hand.

The present study explored the instantaneous effect of embodiment perception on the alteration of neural connectivity. Previous studies indicated that visual inputs induced mirror illusion combined with vibrotactile stimulation evoked kinesthesia illusion could promote embodiment perception *via* multisensory integration and presented an enhancement of embodiment with upgrading activation of the motor region (Wittkopf et al., 2017; Ding et al., 2020a,b). As suggested by the average node degree, dense network connectivity was observed in subjects while perceiving embodiment, and large differences in network connectivity were demonstrated among conditions. This observation indicates the centrality of embodiment perception in the EEG-based network and suggested a tendency of more obvious centrality of strengthening embodiment while receiving the combination of MVF and vibrotactile stimulation. Moreover, subsequent observations of node degrees showed centralities of the embodiment perception mainly in the left central area in the alpha and the beta bands. Similar to our observation, studies reported the activation of the motor cortex during MVF, when subjects had mirror illusion/embodiment (Lee et al., 2015; Ding et al., 2020a). Centralities in the left frontal, bilateral occipital-parietal, and bilateral temporal areas were also obtained in the condition of MVF and vt-MVF in our study. This finding is in line with our previous observations and provides tentative evidence for the instant effects of embodiment on mediating the information transmission in the visual stream (Goodale and Milner, 1992; Ding et al., 2019). The ventral-dorsal visual streams comprised the temporal, parietal, and visual cortex, which play an important role in the perceptual identification of objects and sensorimotor transformations (Goodale and Milner, 1992). In addition, this study demonstrates a trend of prominent centralities in visual stream areas from the beta band to the alpha band while embodiment perception is enhanced *via* the combination of vibrotactile stimulation. Thus, we inferred a positive relationship between embodiment perception and network topology, especially in the visual stream-involved regions.

An efficient network would contribute to the enhancement of human brain functions, which relied on suitable properties of the network (Bassett et al., 2006; Laney et al., 2015). A network with a high clustering coefficient (segregation) and short characteristic path length (integration) was regarded as an optimal network, where the efficiency of local and/or global information communication and processing was facilitated (Bassett et al., 2006). Previous studies reported enhanced neural communication after the intervention of MVF, which indicated the long-term effects of embodiment (Hamzei et al., 2012; Saleh et al., 2014; Ding et al., 2019). In the present study, increased global and local neural communication efficiencies were obtained in the condition of vt-MVF as an instant effect of enhanced embodiment, compared to the other two conditions. To our knowledge, our study is the first to demonstrate the prominent segregation and integration of brain networks in healthy subjects during motor tasks while receiving MVF with vibrotactile stimuli. However, significant alterations of neural communications were only observed in the vt-MVF condition. Our previous study supported that the combination of MVF and vibrotactile stimuli could strengthen the embodiment perception and activate motor regions in both alpha and beta bands (Ding et al., 2020a). Thus, we speculated that the enhanced embodiment *via* multi-sensory integration might have more prominent instant effects on neural communication in healthy subjects. In addition, this study also reported that the enhanced embodiment could generate a shift in global neural communication efficiency toward the dominant side, which might contribute to the practice of the vt-MVF in the clinic for hemiplegic paralysis rehabilitation. Although no significant alterations were found in the condition of MVF, a similar trend was still demonstrated in the beta band when compared to the sham-MVF. One possible interpretation might be the alpha oscillations were the dominant rhythm during wakeful relaxation with closed eyes (Lindgren et al., 1999), while the beta oscillations were more involved in active tasks, including cognitive tasks (Jin et al., 2012). In our study, subjects were required to watch the reflection of their non-dominant hand and feel as if his/her dominant hand was moving with the image in the mirror. Thus, the beta band would be more sensitive and predominant. The MVF-induced embodiment might have a long-term effect on shifting brain function toward an efficient pattern, but it might be difficult or insufficient to observe its instant effect in our study. This might be another potential reason.

It should be noted that opposite results were shown in the theta band, where larger connections and enhanced global brain neural communication efficiency were revealed in healthy subjects without MVF stimulation. Theta rhythm appears during a relaxed brain state and is involved in inward-focused concentration (Shenefelt, 2018), which might be one possible reason. Moreover, motor imagery showed increased theta synchronization, which reflected an increased effort, and reduction of visuospatial attention (Lubbe et al., 2021). Thus, the major involvement of motor imagery in the condition of sham-MVF might contribute to the results. Further studies were necessary to investigate these factors.

## 5. Limitation

As an in-depth analysis of our previous experiment, this study suggested potential mechanisms of MVF from the perspective of brain connectivity. Although this was a pilot study, the small sample size still hindered the power of statistical analyses. Second, it might provide more suggestions and directions for the clinic, if the subjects were elderly healthy people or stroke patients. Third, it would be better to use individualized frequency bands, which might eliminate the impact of interindividual differences in EEG frequencies. Furthermore, the data were collected by a 32-channel wireless EEG cap, which might limit further exploration of sub-network alterations.

## 6. Conclusion

This study explored the instant effects of MVF on brain connectivity for new insight, which revealed the capacity of embodiment perception to strengthen network connectivity and facilitate neural communication efficiency in healthy subjects. Moreover, this study also provided tentative evidence that a stronger embodiment sense induced *via* the combination of mirror visual and vibrotactile stimulus could enhance neural communication efficiency and generate a shift toward the hemisphere of the dominant hand. These findings might contribute to the investigation of the therapeutic effect of MVF and the development of a new rehabilitation strategy.

## Data availability statement

The raw data supporting the conclusion of this article will be made available by the authors, without undue reservation.

## Ethics statement

The studies involving human participants were reviewed and approved by the Research Ethics Committee of the University of Waterloo. The patients/participants provided their written informed consent to participate in this study.

## Author contributions

JJ, JH, and NJ conceived the study. LD performed the experiment. LD and QS analyzed the data and wrote the manuscript. All authors read and approved the final manuscript.

## Footnotes

1
https://www.nitrc.org/projects/bct/
